# Supplementary material for: The Biological Functions and Intestinal Inflammation Regulation of IL-21 in Grass Carp (Ctenopharyngodon idella) during Infection with Aeromonas hydrophila
Source: Cells. 2023 Sep 14;12(18):2276. doi: 10.3390/cells12182276 (PMC10528265; doi:10.3390/cells12182276)
Supplement: Supplementary file 1 [file cells-12-02276-s001.zip › cells-2572534-supplementary.pdf]

Supplementary Material

The biological functions and intestinal inflammation regulation of IL-21 in grass carp *(Ctenopharyngodon idella)* during infection with *Aeromonas hydrophila*

Gaoliang Yuan, Weihua Zhao, Yanwei Zhang, Zhao Jia, Kangyong Chen, Junya Wang, Hao Feng, and Jun Zou

1 Supplementary Figures and Tables

1.1 Supplementary Figures

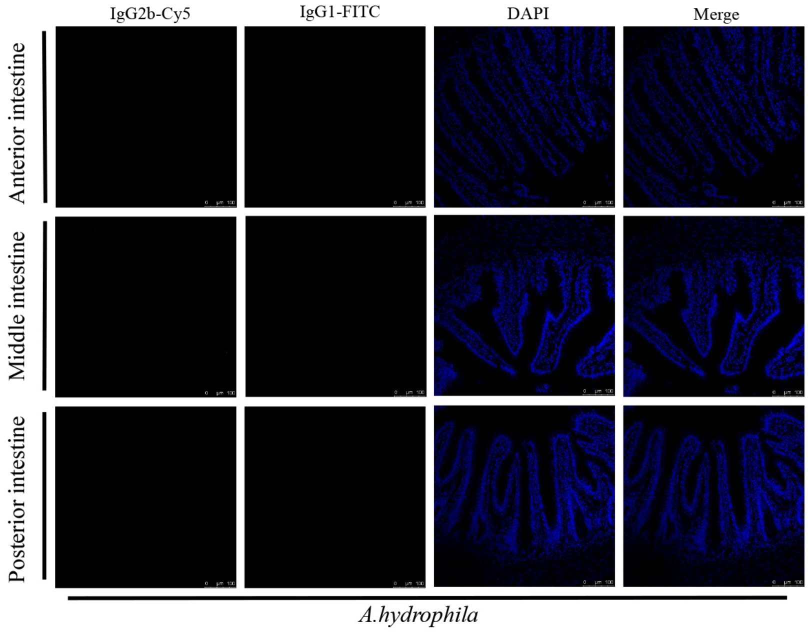

Supplementary Figure S1. Analysis of the IL-21$^+$ cells in anterior, middle and posterior intestine of grass carp by fluorescent microscopy. Fish were i.p. injected with 100 µl of *A. hydrophila* (1×10^7 CFU/ml). After 72 h, anterior, middle and posterior intestine were fixed and then cryosectioned for immunofluorescent staining using isotype antibodies (IgG1-FITC and IgG2b-Cy5) at 4°C overnight. Nucleus (blue) was counterstained with DAPI.

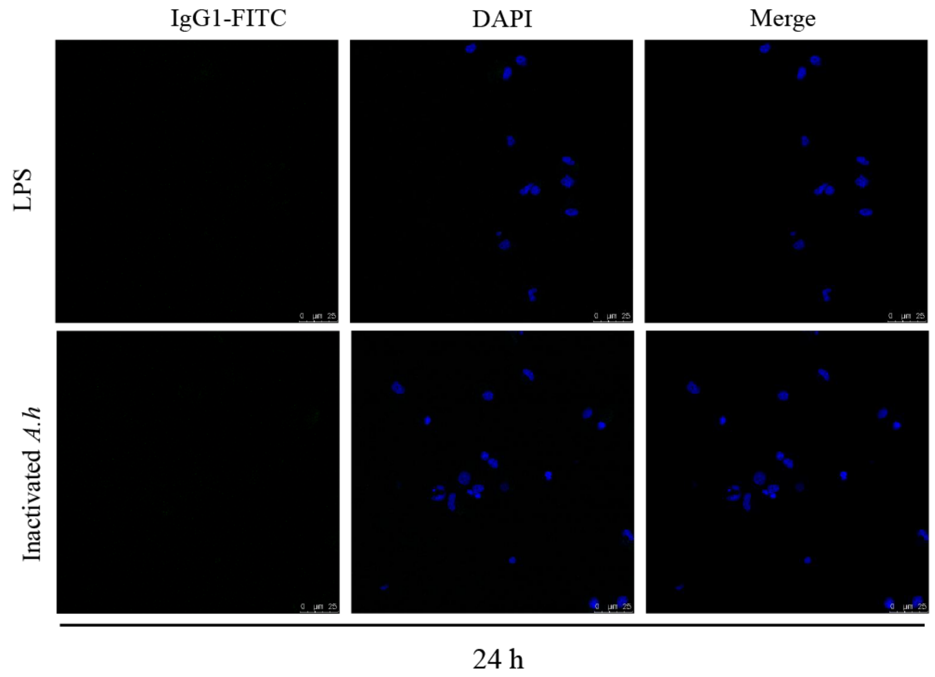

Supplementary Figure S2. Confocal microscopic analysis of IL-21 the primary kidney leukocytes stimulated with LPS or inactivated *A. hydrophila*. The primary kidney leukocytes were immunostaining with isotype antibody (IgG1-FITC) at 4°C overnight. Nucleus (blue) was counterstained with DAPI.

```
                    C/EBPα                         AP-1
CATCTGAGGTGTTTTTGTTCGTTTGTTTGTTTTTTTTTATACTGAAGTATATGAGTCATA   −2000
                             AP-1
AAAGTCATATAGTATACAACAAGTATATAAGTCATGCATCAAATATGATACTCAACAAAA   −1940
   C/EBPα
ACACATATTGCAAACTTTTTTGTGTATGTATGTATATATATATATATATATATTCTGTCT   −1880
AAAGGTTTAACAAACTGTTCCATTTAAAAATTAATCTGAAAATATTCCAGCATATTCCAT   −1820
                   c-Jun
CTAGCCTTCTCATTGGTCCTCATGCTTCACACATCGTTGGGACGTATTTTCATCATCAGC   −1760
                            C/EBPα
CACAGAAAACACAGATCTGTATCAGAGAACTTTTGCATGTTTTATGTATGTCATGATATT   −1700
 CREB          Sp-1
GATGTCACCCCTAGACCCCCTCCCACATCCTCACACATAAACCCACACGCTCATTGTGCT   −1640
     Sp-1                     Sp-1        NF-κB      c-Jun
TTCCAGGTTGAGGGAAACGATTCAGATCTAGACATGACTGACAGAGGAAAGTTTTGAATC   −1580
                                        Sp-1        NF-κB
ATGCCGTTTTAAACTTGAGCACAAATTACTTCCATGATCCTGCCGAAGGGAGGAATCCCC   −1520
                                   NF-κB
AGAGCGAACCTGTCCTGTCTGGGGGTCACTAGGGCCTTCCCCTGCCGCCGTGACACTATG   −1460
                    Sp-1                   Sp-1
GGTATGTCAGCCACCAACCTCCGCCCCCCACTTCCTGTTCAGCGCAGCCCGCGATTGGCC   −1400
 Sp-1                                 NF-κB      C/EBPα
GGCGGAGGGGTTCTTTCACAGGCCCAATGTGATCTCATTTCCTGTTCTATGATGTTGAGA   −1340
GTGATCGCGTCTTTCAAAGTGTGTCGTGACACTGCGGAACTGAAGCCACAAGCTGTGTTG   −1280
                                  C/EBPα
TTTTAGTATTACTGAGATACTATTACGCTGAACACAAACTGCTATTAGTAAAATGCACAA   −1220
                                        C/EBPα
ATAATCACAAAGAAGTAACACATTGAATTAAACGTAAATGAAATGCAAAACATACTCCAA   −1160
      C/EBPα           C/EBPα          C/EBPα
TACATTTTTTTGGTAATATTTATATTTATTTTAATAATATTTATTTTTATTTTATATTGT   −1100
                          C/EBPα              C/EBPα
TAAATTATTATTTTTTAGTGAAAGTTTTGGTAATTTTTAGTAGTTTTTGTTTTGTTTATA   −1040
      C/EBPα
CAGTTTTTAATATTTTCATTTCAGTTTTAGCTTTAATTATTTTAATACATCAAGTTAGAC    −980
                C/EBPα
TAAATGAAAATGAGAAATGATGACAGGGCAAGTAGCAAGTTTATTTTATTTCAAGTAATG    −920
        IRF-1      C/EBPα           C/EBPα
AAAATGTGTTTTGTTTTTATTATTTATTTTATTTTATATATTTTTTTTTATATAGTCATTT    −860
TTAGTAGTTTTTTTTTTTTTTTTTTTACATACGCCTGTAATTTTTTATTTAAGTTTTAGCT    −800
                                                   Sp-1
TATTTTAGTAAGTCAAGTTAGACTAAATGAAAATGAGAAATGATGCCGGGGCAAGTAGCA    −740
AGTTTTTAGATTATATATATATATATATATATAATTTCTTTTATTTCAAGCAATTGTTTT    −680
                                              CREB
AATTTTATCTAACAATAATAACCTTGGCCACAAGCAGGTTTGACGTCCAAACACAGGTCA    −620
                                C/EBPα                C/EBPα
TTTAACACTCTGCAGGTTTTGGGTGCAAATATTTCATTTCCAGGGTCTATTCTTATCTCA    −560
CAAGATGAAAAGTACATTAAGCTTCCTGTTTTTGTCTATAATCAATATGGTTTGTGTGGG    −500
NF-κB
AAGCCGCAAAGTAAAAAGCACCTTTGTCATATCCACTTGCGCTCGATGATGCTGCACCTA    −440
                              AP-1        Sp-1
TAGTCGCACACACATGTTTCTGATGCTGATTCAGAATGAGAAGAGGAACTGACAGCATTT    −380
TGTAGTTTCCTGCCAAGTGTGCAGAAAGTCTGGGTCAGGTTCACACACACACAGAAACAC    −320
ACACACTGCATCCCGTACACCCCCGCGCACGCGCCTCTCGATTACATCTTTGTCAACTGA    −26
CAGTCTGACGAAGGCCAGTGGAAAAGAAAAAGTAAATTACTCATTGTAAAAACTTGACTT    −200
                                  Sp-1
TCCAATTCTCCACAGAGAAGCGGTTTTGCTCCGTCCTCAAGGATACTTATATAAAGGTGA    −140
TTGTGTACCTGTTGACAGTATGAAGGACATCAATCCAACTAGCTTTAGAGAAGGTACAGA    −80
AGAAACTTTACAGCTTAAAC                                            −20
```

Supplementary Figure S3. Nucleotide sequences and putative regulatory elements of grass carp *il21* promoter region. The putative transcription factor binding sites are underlined.

1.2 Supplementary Tables

Supplementary Table S1. Information for gene primers.

Primers	Sequence (5′ to 3′)	Application
***il21*-F**	GTACAGTGGGAAACTAGGGAGC	qRT-PCR
***il21*-R**	GCTCTTCTCTTGGAAATCGAAG	qRT-PCR
***il21*r-F**	GTACAGTGGGAAACTAGGGAGC	qRT-PCR
***il21*r-R**	GCTCTTCTCTTGGAAATCGAAG	qRT-PCR
***glysozyme*-F**	CTGATAGATGGGTGGGGCGA	qRT-PCR
***glysozyme*-R**	GGACCTGCGTTGTAGGCTGA	qRT-PCR
***clysozyme*-F**	GGTTTAAGACCCAAAGAGTGCGT	qRT-PCR
***clysozyme*-R**	AGTAACTATCCCAGGTTTCCCAT	qRT-PCR

***ef1α*-F**	AGCACAAACATGGGCTGGTTC	qRT-PCR
***ef1α*-R**	ACGGGTACAGTTCCAATACCTCCA	qRT-PCR
***il1β*-F**	TCTCCTCGTCTGCTGGGTGT	qRT-PCR
***il1β*-R**	CAAGACCAGGTGAGGGGAAG	qRT-PCR
***il10*-F**	GCAACAGAACATCAATAGTCCTT	qRT-PCR
***il10*-R**	CACCCTTTTCCTTCATCTTTTCA	qRT-PCR
***βdefensin1*-F**	GCATCATTTCCCTGGACCTG	qRT-PCR
***βdefensin1*-R**	AATCCCTTGCCACAGCCTAA	qRT-PCR
***β-defensin2*-F**	GGACTTGTGGCTATGGAGGA	qRT-PCR
***β-defensin2*-R**	CAGCATCTGTATTTGCGTGG	qRT-PCR
***β-defensin3*-F**	TGAAGCCGATGACACAGACAT	qRT-PCR
***β-defensin3*-R**	CACCTGTATCTTCGAGGGCAA	qRT-PCR
***nf-κbp52*-F**	GTCAGTGTAACGACAACGGGA	qRT-PCR
***nfκbp52*-R**	TAGCCTGTTCCTCTGCATCAC	qRT-PCR
***nfκbp65*-F**	GTGCGACAAAGTGCAGAAGG	qRT-PCR
***nfκbp65*-R**	AAACACGATAGCCACCTGCC	qRT-PCR
***il21* promoter-F1**	CCTGAGCTCGCTAGCCTCGACATCTGAGGT GTTTTTGTTCGTTT	Plasmid construction
***il21* promoter-F2**	CCTGAGCTCGCTAGCCTCGACTGCCGCCGT GACACTATG	Plasmid construction
***il21* promoter-F3**	CCTGAGCTCGCTAGCCTCGAAAAGTAAAA AGCACCTTTGTCATATCC	Plasmid construction
***il21* promoter-R**	CAGTACCGGATTGCCAGCTTGTTTAAGCTG TAAAGTTTCTTCTGTACCT	Plasmid construction
